# Single-center analysis of etiology and complications in hospitalized children with chronic kidney disease stages 3–5

**DOI:** 10.3389/fped.2026.1712781

**Published:** 2026-03-27

**Authors:** Ying Liang, Ying Shen, Yeping Jiang

**Affiliations:** Department of Nephrology, Beijing Children’s Hospital, Capital Medical University, Beijing, China

**Keywords:** children, chronic kidney disease, complication, etiology, hospitalization

## Abstract

**Objective:**

To investigate the etiology spectrum and complication profiles of hospitalized children with chronic kidney disease (CKD) stages 3–5, identify independent risk factors for CKD progression, and provide evidence for optimizing early clinical management. We hypothesized that congenital anomalies of the kidney and urinary tract (CAKUT) are the predominant etiology in this cohort, and baseline eGFR decline plus uncontrolled hypertension would independently predict progression to stage 5 CKD.

**Methods:**

A retrospective cohort study was conducted on 170 children with CKD stages 3–5 admitted to the Nephrology Department of Beijing Children's Hospital from January 2016 to December 2023. Etiology, complication rates, and their associations with CKD stages were analyzed. Multivariate logistic regression models were used to identify independent risk factors for progression to stage 5 CKD, adjusting for age, sex, and underlying etiology.

**Results:**

CAKUT accounted for 51.8% of cases, followed by glomerular diseases (22.4%). The prevalence of anemia (80.5%), hypertension (64.1%), and CKD-MBD (90.6%) increased significantly with CKD progression (*P* < 0.001 for all). Anemia occurred in 100% of stage 5 non-dialysis patients, while hypertension control rates were only 45.3%. Multivariate analysis identified baseline eGFR <30 mL/min/1.73 m² (OR = 4.2, 95% CI: 2.1–8.3, *P* < 0.001) and uncontrolled hypertension (OR = 2.5, 95% CI: 1.5–4.2, *P* = 0.001) as independent predictors of progression to stage 5 CKD. The overall infection rate was 28.2%.

**Conclusion:**

CAKUT is the leading etiology of CKD stages 3–5 in hospitalized children, with a high burden of complications. Early intervention for anemia, hypertension, and CKD-MBD, combined with enhanced infection prevention, may improve clinical outcomes. Baseline eGFR <30 mL/min/1.73 m² and uncontrolled hypertension are key targets for delaying CKD progression. Notably, the distribution of etiology and complication burden in this cohort parallels patterns reported in North America and Europe, supporting the global applicability of KDIGO-based management strategies.

## 1 Introduction

The etiology of chronic kidney disease (CKD) in children differs significantly from that in adults, with congenital anomalies of the kidney and urinary tract (CAKUT) and glomerular diseases being the primary causes. Data from North America indicate that obstructive uropathy is the leading subtype of CAKUT, while focal segmental glomerulosclerosis (FSGS) is the most common glomerular disease (1). However, data on the etiology spectrum and complication trends in Chinese pediatric CKD patients—particularly those with advanced stages (3–5)—remain limited, and the independent risk factors for progression in this population require further clarification. This knowledge gap hinders the development of targeted management strategies for Chinese children with advanced CKD.

In the early stages of CKD, complications such as anemia and hypertension may already be present; as the disease progresses, complications including growth retardation, malnutrition, CKD-mineral and bone disorder (CKD-MBD), metabolic acidosis, and cardiovascular diseases become more prominent. These complications not only accelerate CKD progression but also severely impact children's growth, quality of life, and long-term prognosis. Early assessment, identification, and intervention of CKD complications, along with standardized long-term management and monitoring, are therefore crucial.

The present retrospective cohort study aimed to: (1) characterize the etiology spectrum of hospitalized children with CKD stages 3–5 in a single Chinese tertiary pediatric center; (2) analyze the prevalence and stage-specific trends of key complications (anemia, hypertension, CKD-MBD, infections); and (3) identify independent risk factors for progression to stage 5 CKD. We hypothesized that CAKUT would be the predominant etiology, and baseline eGFR decline and uncontrolled hypertension would serve as independent predictors of CKD progression. Our findings indicate that the prevalence patterns and complication burdens observed in this tertiary pediatric nephrology center are consistent with international KDIGO-based data, thereby reinforcing global trends in pediatric CKD.

## 2 Research subjects and methods

### 2.1 Research subjects

This retrospective cohort study included all children (aged 0–18 years) diagnosed with CKD and hospitalized in the Nephrology Department of Beijing Children's Hospital from January 2016 to December 2023. Case ascertainment was performed via electronic medical record (EMR) retrieval using keywords: “chronic kidney disease”, “renal insufficiency”, “CAKUT”, “glomerulopathy”, and “end-stage renal disease”. Inclusion criteria were: (1) diagnosis of CKD stages 3–5 based on KDIGO 2012 guidelines; (2) complete medical records for etiology classification and complication assessment; (3) follow-up data available for progression analysis. Exclusion criteria were: (1) comorbid wasting diseases (malignancies, tuberculosis, severe infectious diseases); (2) major congenital anomalies (complex congenital heart disease) that could independently affect renal function; (3) acute kidney injury superimposed on CKD; (4) incomplete medical records. A total of 170 children were enrolled after applying inclusion and exclusion criteria. Multiple hospitalizations of the same patient were counted as a single case, with data extracted from the first hospitalization during the study period.

### 2.2 CKD staging criteria

CKD Diagnostic Criteria: CKD was diagnosed according to the KDIGO Clinical Practice Guideline for the Evaluation and Management of Chronic Kidney Disease, defined as persistent kidney damage (albuminuria, urinary sediment abnormalities, or structural abnormalities on imaging) or decreased estimated glomerular filtration rate (eGFR) < 60 mL/min/1.73 m^2^ for ≥3 months (2). Albuminuria was assessed via urinary albumin-to-creatinine ratio (ACR), with abnormal albuminuria defined as ACR ≥30 mg/g.

eGFR Calculation and CKD Staging: The eGFR was calculated using the revised Schwartz formula (2009): eGFR (mL/min/1.73 m^2^) = K (age-specific constant) × height (cm)/serum creatinine (Scr, μmol/L). The K-values were defined as follows: 0.33 for preterm infants, 0.45 for term infants to 1 year, 0.55 for ages 2–12 years, 0.55 for females aged 13–18 years, and 0.70 for males aged 13–18 years. CKD staging was based on eGFR values using KDIGO 2012 criteria: stage 3 (30–59 mL/min/1.73 m^2^), stage 4 (15–29 mL/min/1.73 m^2^), and stage 5 (<15 mL/min/1.73 m^2^, including dialysis-dependent patients).

### 2.3 Exclusion criteria

Children with comorbid wasting diseases (malignancies, tuberculosis, severe infectious diseases) or major congenital anomalies (complex congenital heart disease) were excluded.

### 2.4 Diagnostic criteria for complications

#### 2.4.1 Hypertension

Hypertension Diagnostic Criteria: Defined according to the KDIGO 2021 Clinical Practice Guideline for the Management of Blood Pressure in CKD: average systolic blood pressure (SBP) or diastolic blood pressure (DBP) > 95th percentile for gender, age, and height (based on Chinese pediatric blood pressure reference standards), or current use of antihypertensive medication (3). Blood pressure measurement was performed using a standardized electronic sphygmomanometer with age-appropriate cuffs (cuff width covering 40%–50% of the upper arm circumference). Patients rested in the supine position for 5 min before measurement; three consecutive measurements were taken at 1-minute intervals, with the average value recorded. Hypertension control was defined as SBP and DBP ≤95th percentile for gender, age, and height (with or without antihypertensive medication).

#### 2.4.2 Anemia

Anemia Diagnostic Criteria: Diagnosed according to the KDIGO 2012 Clinical Practice Guideline for Anemia in CKD, defined as hemoglobin (Hb) level below the 5th percentile for age and gender (reference standards: Hb <110 g/L for children aged 1–5 years, <115 g/L for 6–11 years, <120 g/L for 12–18 years). Severe anemia was defined as Hb <70 g/L (4).

#### 2.4.3 CKD-mineral and bone disorder (CKD-MBD)

CKD-MBD refers to systemic mineral and bone metabolism disorders caused by CKD progression, including one or more of the following: (1) abnormal calcium, phosphorus, parathyroid hormone (PTH), or vitamin D metabolism; (2) abnormal bone turnover, mineralization, volume, linear growth, or strength; (3) vascular or other soft tissue calcification.

#### 2.4.4 Other key outcomes

Other Key Outcome Definitions: Short stature was defined as height <3rd percentile for age and gender based on the WHO 2007 Child Growth Standards. Malnutrition was diagnosed using the BMI z-score, with malnutrition defined as BMI z-score ≤ 2. Cardiovascular (CV) risk was defined as the presence of one or more of the following: left ventricular hypertrophy (detected by echocardiography), hypertension, hyperphosphatemia, or vascular calcification (detected by computed tomography or echocardiography).

### 2.5 Laboratory measurements

Laboratory Measurements: Serum creatinine was measured using an enzymatic method on a Beckman Coulter AU5800 automatic biochemical analyzer (Beckman Coulter, Inc., Brea, CA, USA), calibrated with traceable standards (NIST SRM 967). Hb was detected via cyanmethemoglobin method. Serum calcium, phosphorus, albumin, and uric acid were measured using standard biochemical assays on the same analyzer. Intact PTH (iPTH) was measured by chemiluminescent immunoassay (Siemens ADVIA Centaur XP, Siemens Healthcare Diagnostics, Inc., Tarrytown, NY, USA). All laboratory tests were performed in the central laboratory of Beijing Children's Hospital, with internal quality control conducted daily and external quality assessment participated quarterly.

### 2.6 Data collection

Detailed medical records were collected for all patients, including hospitalization history, general information (gender, date of birth, height, weight, blood pressure), past medical history (birth history, previous hospitalizations), family history, laboratory tests (blood tests, renal function, electrolytes, PTH), imaging studies (renal ultrasound), and treatment regimens (antihypertensive drugs, iron supplements, erythropoietin, phosphate binders).

### 2.7 Statistical analysis

Statistical Methods (Descriptive Analysis): Statistical analysis was performed using SPSS 26.0 software. Normally distributed continuous variables were expressed as mean ± standard deviation, non-normally distributed data as median [interquartile range (IQR)], and categorical variables as frequency (percentage). Comparisons between two groups were conducted using independent samples t-test (normally distributed data) or Mann–Whitney *U*-test (non-normally distributed data). Multi-group comparisons were performed using one-way analysis of variance (ANOVA) or Kruskal–Wallis H test, followed by *post-hoc* tests as appropriate.

Statistical Methods (Progression Analysis): CKD progression was defined as the transition from stage 3 or 4 to stage 5, including initiation of dialysis, during follow-up. Multivariate logistic regression models were used to identify independent risk factors for progression to stage 5 CKD, with adjustment for potential confounders including age, gender, underlying etiology (CAKUT vs. glomerular disease), and anemia severity. Variables with *P* < 0.1 in univariate analysis were included in the multivariate model. A two-tailed *P*-value <0.05 was considered statistically significant. All participants had relatively uniform follow-up duration (median 38.6 months, IQR 24.3–52.9 months), and the endpoint was binary (progression to stage 5 vs. no progression), which justified the use of logistic regression for progression analysis.The Nagelkerke *R*^2^ was calculated to assess model performance (Nagelkerke *R*^2^ = 0.38), indicating moderate goodness of fit.

## 3 Results

A total of 170 children with CKD stages 3–5 were included, with a median age of 11.34 years (IQR: 8.67–12.92). The majority (64.7%) were aged >10 years and 62.4% were male. CKD stage distribution was as follows: stage 3 (20.6%), stage 4 (15.9%), stage 5 non-dialysis (45.9%), and stage 5 dialysis (17.6%). Among the dialysis patients, 17 (56.7%) were on hemodialysis, and 13 (43.3%) were on peritoneal dialysis. The age distribution was as follows: 0–6 years (26 cases, 15.3%), 6–10 years (34 cases, 20.0%), and >10 years (110 cases, 64.7%). See Table 1 for details.

**Table 1:** General characteristics of hospitalized children with chronic kidney disease (CKD) stages 3–5.

Characteristic	*N* (%) or median (interquartile range, IQR)
Total number of patients	170
Gender: Male	106 (62.4%)
Age, years	11.34 (8.67–12.92)
Age at diagnosis, years	
0–6	26 (15.3%)
6–10	34 (20.0%)
≥10	110 (64.7%)
CKD stage	
Stage 3	35 (20.6%)
Stage 4	27 (15.9%)
Stage 5 non-dialysis	78 (45.9%)
Stage 5 dialysis	30 (17.6%)
Dialysis modality (stage 5 dialysis)	
Hemodialysis	17 (56.7%)
Peritoneal dialysis	13 (43.3%)
Preterm birth (<37 weeks)	13 (7.6%)
Birth weight, g	
<2,500	11 (6.5%)
2,500–4,000	136 (80.0%)
>4,000	18 (10.6%)

Missing data: Birth information for 5 patients from welfare institutions.

Among the 170 children, CAKUT was the leading cause (88 cases, 51.8%), including renal dysplasia/hypoplasia (43 cases, 25.3%), neurogenic bladder (12 cases, 7.1%), polycystic kidney disease (13 cases, 7.6%), obstructive nephropathy (10 cases, 5.9%), solitary kidney/horseshoe kidney (7 cases, 4.1%), vesicoureteral reflux/reflux nephropathy (2 cases, 1.2%), and medullary cystic disease (1 case, 0.6%). Glomerular diseases accounted for 38 cases (22.4%), including primary glomerular diseases (23 cases, 13.5%), with FSGS being the most common (16 cases). Among these primary glomerular disease cases, 14 (60.9%) were already in end-stage renal disease (ESRD) or on renal replacement therapy at the time of diagnosis. Secondary renal diseases accounted for 15 cases (8.8%), including ANCA-associated vasculitis (9 cases, 5.3%), lupus nephritis (2 cases, 1.2%), Henoch-Schönlein purpura nephritis (2 cases, 1.2%), and hemolytic uremic syndrome (2 cases, 1.2%). Hereditary kidney diseases accounted for 9 cases (5.3%), and other diseases accounted for 6 cases (3.5%). The etiology was unknown in 29 cases (17.1%).

Analysis of Unknown Etiology Cases: Among the 29 cases with unknown etiology, 19 (65.5%) were already in ESRD at diagnosis, accounting for 17.6% of all ESRD cases in the cohort. This high proportion may be attributed to late referral: 21 (72.4%) of these 29 cases had no prior history of pediatric nephrology follow-up before hospitalization, and non-specific clinical manifestations in ESRD further hindered etiological diagnosis. Notably, 8 (27.6%) of the unknown etiology cases underwent genetic testing, but no pathogenic mutations were identified.

The 170 children were divided into two groups based on the reason for their first visit: asymptomatic laboratory findings (32 cases, 18.8%) and symptomatic onset (138 cases, 81.2%). In the asymptomatic group, CKD staging was as follows: stage 3 (34.4%), stage 4 (28.1%), and stage 5 (37.5%). In the symptomatic group, CKD staging was as follows: stage 3 (17.4%), stage 4 (13.0%), and stage 5 (69.6%). The difference in CKD staging between the two groups was statistically significant (*P* = 0.001), indicating a correlation between the reason for the first visit and CKD staging. In the asymptomatic group, 37.6% were already in ESRD at diagnosis, while in the symptomatic group, 69.6% were in ESRD. Table 2 shows the distribution of CKD staging in asymptomatic and symptomatic groups.

**Table 2:** Distribution of CKD stages by initial presentation (asymptomatic vs. symptomatic).

Group	CKD stage 3, *n* (%)	CKD stage 4, *n* (%)	CKD stage 5, *n* (%)	Total, *n* (%)
Asymptomatic (incidental finding)	11 (34.4%)	9 (28.1%)	12 (37.5%)	32 (100%)
Symptomatic	24 (17.4%)	18 (13.0%)	96 (69.6%)	138 (100%)
*P*-value				0.001

Infections occurred in 48 patients (28.2%), predominantly respiratory infections (52.1%), urinary tract infections (22.9%), and catheter-related infections (14.6%). Infection rates escalated with CKD progression: stage 3 (12.0%), stage 4 (18.5%), stage 5 non-dialysis (24.4%), and stage 5 dialysis (43.3%). The most common pathogens were Streptococcus pneumoniae (respiratory infections) and Escherichia coli (urinary tract infections). Table 3 shows laboratory parameters stratified by CKD stage.

**Table 3:** Laboratory parameters stratified by CKD stage.

Parameter	CKD Stage 3 (*n* = 35)	CKD Stage 4 (*n* = 27)	CKD Stage 5 Non-Dialysis (*n* = 78)	CKD Stage 5 Dialysis (*n* = 30)	*P*-value
Hemoglobin (g/L)	131.0 (110.0–141.0)	94.0 (82.0–117.0)	77.0 (63.75–91.0)	91.5 (71.0–106.3)	<0.001
Serum creatinine (μmol/L)	109.9 (83.7–130.9)	237.1 (180.0–293.6)	655.5 (397.2–2016.8)	760.0 (584.2–965.4)	<0.001
Blood urea nitrogen (mmol/L)	8.47 (6.99–11.76)	18.29 (15.12–23.48)	34.08 (22.15–50.82)	28.28 (19.05–32.38)	<0.001
Uric acid (μmol/L)	467.49 ± 124.08	502.33 ± 166.82	508.04 ± 181.70	474.07 ± 163.35	0.626
Serum albumin (g/L)	40.7 (35.9–43.4)	40.8 (37.1–44.6)	36.6 (29.8–42.0)	36.3 (31.9–40.8)	0.006
Prealbumin (g/L)	280.37 ± 67.98	251.48 ± 49.90	287.50 ± 86.24	298.27 ± 53.10	0.044
Alkaline phosphatase (U/L)	192.0 (140.0–300.0)	251.0 (124.0–345.0)	252.5 (111.8–436.0)	179.5 (119.5–295.75)	0.316
Transferrin (g/L)	2.13 (1.89–2.40)	1.93 (1.63–2.10)	1.77 (1.49–2.08)	1.66 (1.44–1.88)	<0.001
Serum calcium (mmol/L)	2.45 (2.30–2.52)	2.36 (2.17–2.44)	1.83 (1.46–2.25)	2.23 (1.97–2.44)	<0.001
Serum phosphorus (mmol/L)	1.62 ± 0.33	1.98 ± 0.65	2.55 ± 0.85	2.29 ± 0.91	<0.001
Intact parathyroid hormone (iPTH, pg/mL)	44.2 (29.2–58.1)	168 (93.4–293.9)	612.7 (286.0–941.1)	290.8 (181.3–704.5)	<0.001

Data presented as median (IQR) for non-normally distributed variables and mean ± standard deviation for normally distributed variables.

Anemia prevalence increased significantly with CKD progression: stage 3 (37.1%), stage 4 (70.4%), stage 5 non-dialysis (100%), and stage 5 dialysis (90.0%) (*P* < 0.001). Severe anemia (Hb < 70 g/L) was observed in 8.0% of cases, predominantly in advanced stages (10.3% in stage 5 non-dialysis). Anemia severity correlated with reduced quality of life and increased hospitalization rates. Hemoglobin levels decreased significantly from stage 3 to stage 5 (*P* = 0.003). Among dialysis patients, hemoglobin levels were slightly higher than in non-dialysis patients, but the difference was not statistically significant (*P* = 0.051). The use of iron supplements and erythropoietin (EPO) increased significantly from stage 3 to stage 5: oral iron use (61.5% in stage 3 vs. 88.5% in stage 5 non-dialysis) and EPO use (15.4% in stage 3 vs. 70.4% in stage 5 dialysis). See Table 4 for details.

**Table 4:** Prevalence and management of anemia by CKD stage.

Parameter	CKD stage 3 (*n* = 13)	CKD stage 4 (*n* = 19)	CKD stage 5 non-dialysis (*n* = 78)	CKD stage 5 dialysis (*n* = 27)	Total (*n* = 137)
Anemia severity, *n* (%)					
Severe anemia (Hb <70 g/L)	0 (0.0%)	1 (5.3%)	8 (10.3%)	2 (7.4%)	11 (8.0%)
Moderate anemia (Hb 70–99 g/L)	2 (15.4%)	10 (52.6%)	48 (61.5%)	12 (44.4%)	72 (52.3%)
Mild anemia (Hb ≥100 g/L)	11 (84.6%)	8 (42.1%)	21 (26.9%)	13 (48.9%)	53 (38.7%)
Hemoglobin (g/L), mean ± SD	101.0 ± 12.04	85.4 ± 14.95	76.7 ± 19.65	85.3 ± 18.74	81.93 ± 19.54
Treatment, *n* (%)					
Oral iron supplementation	8 (61.5%)	15 (78.9%)	69 (88.5%)	20 (74.1%)	112 (84.8%)
Erythropoietin (EPO) use	2 (15.4%)	8 (42.1%)	46 (59.0%)	19 (70.4%)	75 (54.7%)

Hypertension overall prevalence was 64.1%, escalating from stage 3 (40.0%) to stage 5 dialysis (80.0%) (*P* = 0.001). Poor blood pressure control (only 45.3% achieved target SBP) was linked to accelerated eGFR decline (OR = 1.8, 95% CI: 1.2–2.7, *P* = 0.006).

The most commonly used antihypertensive drugs were angiotensin-converting enzyme inhibitors (ACEIs, 38.2%), angiotensin receptor blockers (ARBs, 29.4%), and calcium channel blockers (CCBs, 22.3%); 10.1% of patients required combined therapy (Table 5).

**Table 5:** Prevalence of complications and antihypertensive medication use by CKD stage.

Parameter	CKD Stage 3 (*n* = 35)	CKD Stage 4 (*n* = 27)	CKD Stage 5 Non-Dialysis (*n* = 78)	CKD Stage 5 Dialysis (*n* = 30)	Total (*n* = 170)	*P*-value
Complications, *n* (%)						
Hypertension	14 (40.0%)	19 (70.4%)	68 (87.2%)	24 (80.0%)	105 (64.1%)	0.001
Controlled hypertension	10 (71.4%)	12 (63.2%)	30 (44.1%)	13 (54.2%)	55 (45.3%)	0.032
CKD-mineral and bone disorder (CKD-MBD)	20 (57.1%)	24 (88.9%)	76 (97.4%)	27 (90.0%)	147 (90.6%)	<0.001
Hyperphosphatemia	21 (60.0%)	20 (74.1%)	71 (91.0%)	25 (83.3%)	137 (80.6%)	0.001
Hypocalcemia	2 (5.7%)	2 (7.4%)	51 (65.4%)	9 (30.0%)	64 (37.6%)	<0.001
Infection	4 (12.0%)	5 (18.5%)	19 (24.4%)	13 (43.3%)	41 (28.2%)	0.008
Antihypertensive medication, *n* (%)						
Angiotensin-converting enzyme inhibitors (ACEIs)	5 (35.7%)	7 (36.8%)	26 (38.2%)	9 (37.5%)	47 (38.2%)	0.987
Angiotensin receptor blockers (ARBs)	4 (28.6%)	5 (26.3%)	19 (27.9%)	8 (33.3%)	36 (29.4%)	0.921
Calcium channel blockers (CCBs)	3 (21.4%)	4 (21.1%)	15 (22.1%)	7 (29.2%)	29 (22.3%)	0.865
Combined therapy	1 (7.1%)	2 (10.5%)	7 (10.3%)	3 (12.5%)	13 (10.1%)	0.943

Controlled hypertension: systolic/diastolic blood pressure ≤95th percentile for age, gender, and height.

Hyperphosphatemia: serum phosphorus >1.8 mmol/L (children <12 years) or >1.6 mmol/L (children ≥12 years).

Hypocalcemia: serum calcium <2.1 mmol/L.

Combined therapy: use of two or more classes of antihypertensive medications.

The overall prevalence of CKD-MBD was 90.6%. The prevalence of hypocalcemia increased with CKD progression: stage 3 (5.7%), stage 4 (7.4%), stage 5 non-dialysis (65.4%), and stage 5 dialysis (30.0%). Hyperphosphatemia was present in 80.6% of cases, with prevalence as follows: stage 3 (60.0%), stage 4 (74.1%), stage 5 non-dialysis (91.0%), and stage 5 dialysis (83.3%). The difference was statistically significant (*P* = 0.001). CKD-MBD was characterized by hyperphosphatemia (80.6%) and secondary hyperparathyroidism (58.2%). Hyperphosphatemia independently predicted cardiovascular risk (OR = 2.1, 95% CI: 1.4–3.2, *P* = 0.002). iPTH levels increased significantly from stage 3 to stage 5 non-dialysis (*P* < 0.001) but decreased in stage 5 dialysis patients (*P* < 0.001). Secondary hyperparathyroidism was present in 17.1% of stage 3, 70.4% of stage 4, 76.9% of stage 5 non-dialysis, and 46.7% of stage 5 dialysis patients. Bone biopsy was not performed in any of the 170 children due to its invasive nature and parental refusal. Among the 42 children who underwent imaging, 10 showed bone structure changes (osteopenia/osteoporosis), and 1 dialysis patient had soft tissue calcification. No cardiac valve calcification was found on echocardiography.

A logistic regression model identified the following independent predictors of progression to stage 5 CKD: Baseline eGFR <30 mL/min/1.73 m^2^ (OR = 4.2, 95% CI: 2.1–8.3, *P* < 0.001), uncontrolled hypertension (OR = 2.5, 95% CI: 1.5–4.2, *P* = 0.001), and serum phosphorus >2.1 mmol/L (OR = 1.9, 95% CI: 1.1–3.3, *P* = 0.02). In contrast, CAKUT etiology was associated with slower progression compared to glomerular disease (OR = 0.6, 95% CI: 0.4–0.9, *P* = 0.04). Additionally, multivariate linear regression showed that lower hemoglobin levels were independently associated with higher iPTH (β = −0.34, 95% CI: −0.51 to −0.17, *P* = 0.001) and elevated phosphorus (β = −0.28, 95% CI: −0.42 to −0.14, *P* = 0.003).

Confidence Intervals for Key Findings: Anemia prevalence by stage: Stage 5 non-dialysis (100%, 95% CI: 95.2%–100%), Stage 5 dialysis (90.0%, 95% CI: 78.6–96.7%). Hypertension risk: Adjusted OR for stage 5 vs. stage 3: 5.8 (95% CI: 3.1–10.9, *P* < 0.001). CKD-MBD predictors: Hypocalcemia (OR = 3.4, 95% CI: 1.8–6.5, *P* < 0.001).

## 4 Discussion

This single-center retrospective cohort study characterized the etiology spectrum and complication profiles of 170 hospitalized children with CKD stages 3–5, identified independent risk factors for progression to stage 5 CKD, and supplemented key gaps in regional pediatric CKD data. Consistent with our hypothesis, CAKUT was the predominant etiology, and baseline eGFR <30 mL/min/1.73 m^2^ plus uncontrolled hypertension emerged as independent predictors of CKD progression. These findings align with global trends in pediatric CKD, with the distribution of etiology and complication burden in this Chinese tertiary pediatric center paralleling patterns reported in North America and Europe, thereby supporting the global applicability of KDIGO-based management strategies.While time-to-event modeling could provide additional granularity in assessing CKD progression, it was not central to the primary research question of identifying independent predictors of progression to stage 5 CKD.

Consistent with global data, CAKUT (51.8%) and glomerular diseases (22.4%) dominated the etiology of pediatric CKD in this cohort (1, 5). This pattern is consistent with international registries, where CAKUT is universally recognized as the leading cause of pediatric CKD, followed by glomerular diseases. The predominance of CAKUT over glomerular causes in our cohort reflects a trend consistent with global observations, likely attributable to advancements in prenatal screening and improved healthcare access. Structural anomalies such as renal hypoplasia (25.3%) and neurogenic bladder (7.1%) were prevalent among CAKUT cases, many of which are surgically correctible if identified early. For example, children with vesicoureteral reflux or obstructive uropathy (5.9%) could benefit from timely interventions like antibiotic prophylaxis or surgical diversion to prevent recurrent infections and scarring.

Among the 38 cases of glomerular diseases, 23 were primary glomerular diseases, with FSGS being the most common (16 cases). FSGS is known for its poor clinical response to immunosuppressive therapy and unfavorable prognosis, consistent with global reports (6). Notably, 60.9% of primary glomerulopathy cases presented at ESRD, highlighting the aggressive nature of these diseases in children. In cases of secondary glomerulonephritis, 73.3% (11/15) had already reached ESRD at diagnosis, emphasizing the need for close renal function monitoring in children with systemic diseases (e.g., ANCA-associated vasculitis, lupus nephritis) to enable early CKD diagnosis and intervention.

The 17.1% unknown etiology rate is a notable finding, with 65.5% of these cases presenting at ESRD. Late referral (72.4% without prior pediatric nephrology follow-up) and non-specific clinical manifestations in ESRD are key contributing factors. This highlights a critical gap in pediatric CKD care in China: asymptomatic children with early-stage CKD are often undiagnosed until advanced stages. With ongoing advancements in genetic testing, more cases of previously undiagnosed hereditary nephropathies may be identified; in our cohort, genetic testing was underutilized, which may have contributed to the unknown etiology rate.

CKD progression was slower in CAKUT patients compared to those with glomerular diseases (OR = 0.6, *P* = 0.04), a phenomenon attributed to preserved tubular function and delayed onset of proteinuria in structural anomalies (5). This finding is consistent with international observations, where CAKUT is often associated with a more indolent progression compared to glomerular diseases. This protective effect underscores the need for aggressive early management of CAKUT to prolong renal survival. For children with unexplained growth retardation or hypertension, renal ultrasound and functional biomarkers should be prioritized, even in the absence of overt symptoms.

In contrast, glomerular diseases, particularly FSGS (16 cases), were associated with rapid progression to ESRD, with 60.9% (14/23) of primary glomerulopathy cases presenting at ESRD. This aligns with global reports of FSGS as a leading cause of pediatric ESRD due to its resistance to immunosuppressive therapy and high recurrence post-transplant (6). Genetic testing for steroid-resistant nephrotic syndrome, as recommended in KDIGO 2024 guidelines, could guide personalized therapies in our cohort.

CKD in children presents with diverse clinical symptoms. In this group, 81.2% of cases presented with symptoms and at diagnosis, 80.5% had anemia, 43.4% had short stature, and 64.1% had hypertension. These findings emphasize the need for renal function evaluation in children with these symptoms. For children with recurrent or severe anemia, unexplained short stature, or hypertension, CKD should be considered, and renal function tests and urological ultrasounds should be performed to enable early detection.

Anemia affected 80.5% of children, escalating to 100% in stage 5 non-dialysis patients. These rates exceed those reported in high-income countries, where early ESA use and iron supplementation achieve higher Hb targets (7). Our multivariate analysis identified independent associations between anemia severity and elevated iPTH (β = −0.34, *P* = 0.001) and hyperphosphatemia (β = −0.28, *P* = 0.003), suggesting that CKD-MBD exacerbates erythropoietic resistance. Despite high rates of iron supplementation (84.8%) and ESA administration (54.7%), median Hb levels remained suboptimal (81.93 ± 19.54 g/L), highlighting gaps in management. These findings are consistent with KDIGO 2012 guidelines, which emphasize proactive ESA administration in stage 3–4 CKD (when Hb <110 g/L) and iron supplementation targeting ferritin >100 ng/mL and transferrin saturation (TSAT) > 20% to prevent severe anemia.

Hypertension prevalence (64.1%) and poor control rates (45.3% achieving target SBP) mirror global challenges in pediatric CKD. Uncontrolled hypertension independently predicted rapid eGFR decline (OR = 1.8, *P* = 0.006). Alarmingly, 34.9% of hypertensive children received no RAAS inhibitors, despite their proven renoprotective and cardioprotective benefits. These findings support the prioritization of ACEIs/ARBs as first-line therapy, even in normotensive children with proteinuria, to reduce glomerular hyperfiltration. Dosing must be titrated to the maximum tolerated dose, as subtherapeutic dosing was common in our cohort.

CKD-MBD affected 90.6% of children, with hyperphosphatemia (80.6%) and secondary hyperparathyroidism (58.2%) as dominant features.The KDIGO 2017 update advocates for balanced management of calcium, phosphorus, and iPTH, yet our data reveal persistent challenges: 76.9% of stage 5 non-dialysis patients had iPTH levels exceeding target ranges (8). In our cohort, only 10 stage 5 patients received sevelamer (a phosphate binder); expanding its use in earlier stages may mitigate hyperphosphatemia. These findings support the use of active vitamin D (calcitriol) as cornerstone therapy, while paricalcitol (a selective vitamin D receptor activator) may offer superior iPTH suppression with less hypercalcemia risk in advanced CKD (9). Bone biopsy, the gold standard for diagnosing renal osteodystrophy, was not performed due to parental refusal, which limits our ability to classify CKD-MBD subtypes (10).

Infections occurred in 28.2% of cases, with rates escalating with CKD progression (43.3% in stage 5 dialysis). This aligns with global pediatric dialysis cohorts (25%–35%) and is attributed to dialysis catheter use, immunosuppression, and nutritional deficits (11). These findings support regular monitoring of immune markers, administration of age-appropriate vaccines, and strict catheter care protocols to reduce infection risk.

This study has several limitations. First, its single-center, retrospective design may introduce selection bias, and the findings may not be generalizable to all Chinese pediatric CKD patients. Second, mortality data were not collected, which limits the assessment of long-term outcomes. Third, bone biopsies were not performed, precluding definitive classification of CKD-MBD subtypes. Fourth, metabolic acidosis was not systematically assessed, which may have underestimated CKD-MBD severity. Fifth, the unknown etiology rate (17.1%) could be reduced with expanded genetic testing. Future prospective, multi-center studies should address these limitations, include mortality data, and explore the impact of socioeconomic factors on CKD progression in China.

## Data Availability

The original contributions presented in the study are included in the article/Supplementary Material, further inquiries can be directed to the corresponding author.
